# Conflicting results between V/Q SPECT and angioscan in pulmonary embolism: what to do?

**DOI:** 10.1186/cc12168

**Published:** 2013-03-19

**Authors:** JM Martel, MN Nadeau, RD Daoust, JP Paquet, JC Chauny

**Affiliations:** 1 Hôpital du Sacré-Coeur de Montréal, Canada

## Introduction

The objective of this study is to determine the final clinical diagnosis of patients who underwent the double investigation for pulmonary embolism with conflicting results. Pulmonary embolism is a prevalent pathology in the emergency department (ED). A certain proportion of patients undergo a double radiological investigation (V/Q SPECT and angioscan), which incurs higher costs and X-ray doses. However, no study to date has addressed this issue.

## Methods

This retrospective study included patients who underwent a double investigation in the ED of Hôpital du Sacré-Coeur de Montréal from April 2008 to October 2012. Patients were selected from a computerized database of medical prescriptions (MediaMed Technologie). Data were then extracted from the patient's files: patient characteristics, radiology report, diagnosis and treatment. Descriptive statistics were conducted.

## Results

In all, 125 patients underwent the double investigation. Our sample had a mean age of 63.1 years (SD ±18.6) and was composed of 82 (65.6%) women. One hundred and fifteen patients (92%) underwent the V/Q SPECT first. The result of 66 (52.8%) SPECT was intermediate or indeterminate. The final diagnosis was pulmonary embolism for 23 patients (18.4%). A significant proportion of patients (19, 38.0%) had conflicting results with the two tests. In this subpopulation, four (21.1%) had a final diagnosis of pulmonary embolism. In the 16 patients with a result of high probability V/Q SPECT and negative angioscan, one (6.3%) had a final diagnosis of pulmonary embolism, but three (100%) with low probability SPECT and positive angioscan were given this final diagnosis.

## Conclusion

Most patients underwent the double investigation because of intermediate or indeterminate V/Q SPECT results. In case of conflicting results, clinicians based their decision on the angioscan. In future studies, it would be useful to identify contributing factors for this discordance.

